# Inactivation of *Schistosoma* Using Low-Temperature Plasma

**DOI:** 10.3390/microorganisms9010032

**Published:** 2020-12-24

**Authors:** Silvie Hejzlarová, Marta Chanová, Josef Khun, Jaroslav Julák, Vladimír Scholtz

**Affiliations:** 1 Department of Physics and Measurements, Faculty of Chemical Engineering, University of Chemistry and Technology, Technická 5, 166 28 Praha, Czech Republic; sisahej@seznam.cz (S.H.); Josef.Khun@vscht.cz (J.K.); 2 Institute of Immunology and Microbiology, 1st Faculty of Medicine, Charles University and General University Hospital in Prague, Studničkova 7, 128 00 Praha 2, Czech Republic; Marta.Chanova@lf1.cuni.cz (M.C.); jaroslav.julak@lf1.cuni.cz (J.J.)

**Keywords:** non-thermal plasma, decontamination

## Abstract

The inactivation of *Schistosoma mansoni* cercariae and miracidia was achieved by exposure to plasma produced by the positive, negative, and axial negative corona discharges. The positive discharge appeared as the most effective, causing the death of cercariae and miracidia within 2–3 min of exposure. The negative discharge was less effective, and the axial discharge was ineffective. The water pre-activated (PAW) by the discharges showed similar efficiency, with the exception of the significantly effective PAW activated with axial discharge. These facts, together with the observation of various reactions among plasma-damaged schistosomes, suggest that the mechanisms of inactivation by different types of discharges are different.

## 1. Introduction

### 1.1. Genus Schistosoma

The genus *Schistosoma* belongs to the phylum Platyhelminthes, class Trematoda, and family Schistosomatidae. This genus contains almost 30 species, and among them, the species *Schistosoma haematobium*, *S*. *mansoni*, *S*. *japonicum*, *S. intercalatum*, and *S*. *mekongi* are important in human medicine as the causative agents of a disease called schistosomiasis or bilharziasis. About 250 million people suffer from schistosomiasis, mainly in Africa, Asia, and South America. In the temperate zone, mostly imported infections occur; however, also spreading from endemic areas with subsequent life-cycle establishment has been recorded [1,2]. Besides schistosomiasis, also cercarial dermatitis or swimmer’s itch, a benign disease of worldwide distribution caused by schistosomes of bird-specific species (e.g., *Trichobilharzia*) occurs frequently also in the temperate zone.

**Figure 1:**
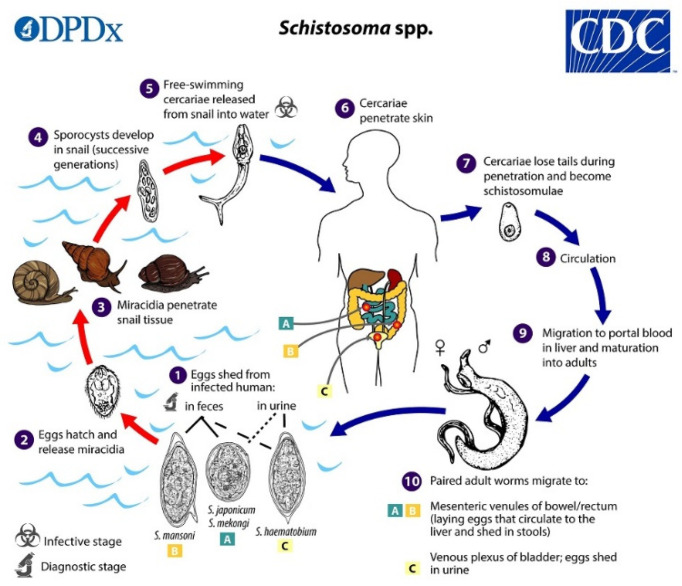
Schematic life-cycle of Schistosome spp. Contributed by the CDC/Alexander J. da Silva, PhD; Melanie Moser https://www.cdc.gov/parasites/schistosomiasis/biology.html.

The life-cycle of schistosomes involves two hosts, namely aquatic snails and mammals. In water, motile miracidia (ciliated larvae) hatch from schistosome eggs, search for the snail, and penetrate its body surface. In snails, miracidia transform into the sporocysts, from which cercariae develop in the snail hepatopancreas and are released from the snail to the water environment. Swimming cercariae are attracted to the skin of vertebrates by chemotaxis and the temperature gradient. During penetration through the skin, they transform into the schistosomula and subsequently enter the blood vessels. In the bloodstream of the host, schistosomes migrate, mature, and lay eggs, which are excreted with urine or feces into water, thus closing the cycle. The life-cycle is schematically shown in Figure 1; for a more detailed description, see, e.g., [3].

Human infection occurs mainly after exposure in natural freshwater reservoirs. Acute and chronic schistosomiasis may be recognized. Initially, Katayama syndrome including fever, headache, myalgia, rash, respiratory symptoms, and hematuria or hematochezia may develop; later on, chronic manifestations develop, including constipation, diarrhea, dysuria, chronic bowel inflammation with intestinal wall ulceration, fibrosis, hyperplasia, polyposis, and hepatosplenomegaly associated with fibrosis and portal hypertension; bladder cancer may develop. A reliable diagnostic method is microscopic detection of eggs in urine, feces, or tissue biopsy together with the detection of specific antibodies, e.g., by ELISA; less frequently, detection of parasitic DNA and RNA by PCR is done [4,5]. Effective peroral medication with praziquantel (PZQ) and oxamniquine (OXA) is available; the mechanism of their action is not known in detail [6].

Besides medication including mass drug administration campaigns, various approaches are used to eradicate schistosomiasis from the environment, among them the improvement of sanitation, intermediate host control, and water body engineering. An effective inactivation of swimming infective schistosome larvae (cercariae and/or miracidia) would be highly appreciated.

Several methods have already been proposed for inactivating schistosomes. Inactivation of cercariae is difficult; variable survival times were reported: according to [7], they can survive up to 36 h in water due to glycogen stores. Temperatures above 45 °C are lethal to cercariae, but at lower temperatures, they survive for up to 100 h [8]. Cercariae survive in distilled water; their viability increases with increasing glucose concentration; on the contrary, higher concentrations of sodium chloride are not favorable to them [9].

Schistosomes can be removed from water by filtration through sand filters [10] or inactivated by chlorination of water [11]. UV radiation in the wavelength range of 200–300 nm has also been used to inactivate schistosomes; cercariae were damaged already when exposed to UV radiation of 3 mL cm^−2^ [12].

### 1.2. Non-Thermal Plasma 

Plasma, called also the fourth state of matter, is a partially or fully ionized gas. There is a distinction between high temperature plasma, reaching temperatures of thousands of Kelvin, and the non-thermal plasma (NTP), which occurs at nearly ambient temperature and contains low-temperature ions and highly energetic free electrons. NTP may be easily obtained by various electric discharges; briefly, the most commonly used are corona discharges, plasma jet (called also plasma needle, plasma torch or plasma pen), dielectric barrier discharge, gliding arc and microwave discharges. For a more detailed description of plasma sources, see, e.g., [13,14,15,16,17,18,19,20]. 

NTP is widely used in many areas of human activity including modification of surface of various materials including nanoporous membranes, in food industry, biotechnology or wastewater treatment. Its applications in biology and medicine summarized numerous reviews [21,22,23,24] or the comprehensive book of [25]. Medical applications include mainly disinfection processes, but also acceleration of the blood coagulation and improved wound healing, dental applications or cancer therapy [26,27]. In addition to the direct action of plasma, the effects of plasma activated water (PAW) persistent for many months after exposure due to the presence of stable reactive oxygen and nitrogen species (RONS) are also significant [28,29,30].

The nature of chemical reactions in NTP is rather complex, for details see [31,32,33]. Various species as ions, radicals, and stable or unstable electroneutral molecules, namely superoxide anion, singlet oxygen, hydroxyl and hydroperoxyl radical, nitric oxide radical, peroxynitrite and others are present. The lifetimes of these species are very short with typical half-periods of life from nanoseconds to a few seconds. The stable compounds formed are hydrogen peroxide, ozone and nitrogen oxides NO_x_. The microbicidal activity of NTP is mediated mainly by RONS arising from surrounding gases, but the mechanisms of biological effects of NTP in unicellular microbes are still poorly understood: Apart from physical destruction, apoptosis also occurs in unicellular microbes including yeasts [34]. Some hallmarks of apoptosis were also found in the higher unicellular eukaryotes, as *Trypanosoma* spp. or *Dictyostelium discoideum* exposed to NTP. 

Most studies on the disinfection effects of NTP have been devoted to bacteria, but attempts to inactivate viruses or fungi both in vitro and in vivo are also frequent; however, a very wide range of experimental parameters and specific results were reported [35,36]. In general, different microbes exhibited different sensitivity to NTP: while bacteria can be completely inactivated in seconds to minutes, yeasts required exposure for several minutes, but mold and bacterial spores for several tens of minutes. Compared to planktonic forms, microorganisms embedded in biofilm are significantly more resistant to the microbicidal action of plasma and therefore require a longer exposure time to achieve inactivation [24].

Reports on the inactivation of parasites using NTP are rather rare. Hayes et al. [37] evaluated a pulsed-gas plasma discharge (PPGD) system for its ability to inactivate the waterborne enteroparasite *Cryptosporidium parvum*, causing cryptosporidiosis in humans. Rowan [38] summarized the broader context of water purification using pulsed-gas plasma, including inactivation of *Cryptosporidium*. Heaselgrave et al. [39] described the inactivation of *Acanthamoeba*, which causes a potentially blinding keratitis most commonly seen in contact lens wearers. Using the system of ambient air plasma confined to the surface of a metallic mesh used as the ground electrode, trophozoites of *Acanthamoeba* were completely inactivated within 0.5–2 min, and complete inactivation of the highly resistant cyst stage was achieved after 4 min of exposure.

To date, only one study has been published on the effect of low-temperature plasma on schistosomes [40]. The dielectric barrier discharge in the stream of carrier gas (He, O_2_, or air) was used as the plasma source. Cercariae of *Schistosoma japonicum* floating on a water suspension surface were exposed, and the damaged or inactivated cercariae sank to the bottom and were evaluated microscopically. Exposure in an oxygen stream has been shown to be effective, but less effective in an air stream; exposure in a helium stream was ineffective. However, in no case the inactivation of all cercaria was achieved even after ten minutes of exposure. The action of RONS is proposed as a mechanism of inactivation: reactive oxygen species cause damage to the surface of the cercariae, diffuse through the cell membrane, and interact with intracellular components that affect the metabolism of the cercariae. Reactive nitrogen particles, especially nitric oxide, are toxic, cause nitration of DNA and proteins, and together with superoxide or hydrogen peroxide, oxidize lipids in the cell wall of cercariae.

In this study, we tried to verify *Schistosoma* inactivation using another source of plasma (different types of corona discharge) and another species (*Schistosoma mansoni*), as well as to supplement the study of cercariae with the study of miracidia.

## 2. Materials and Methods

### 2.1. Corona Discharges

**Figure 2:**
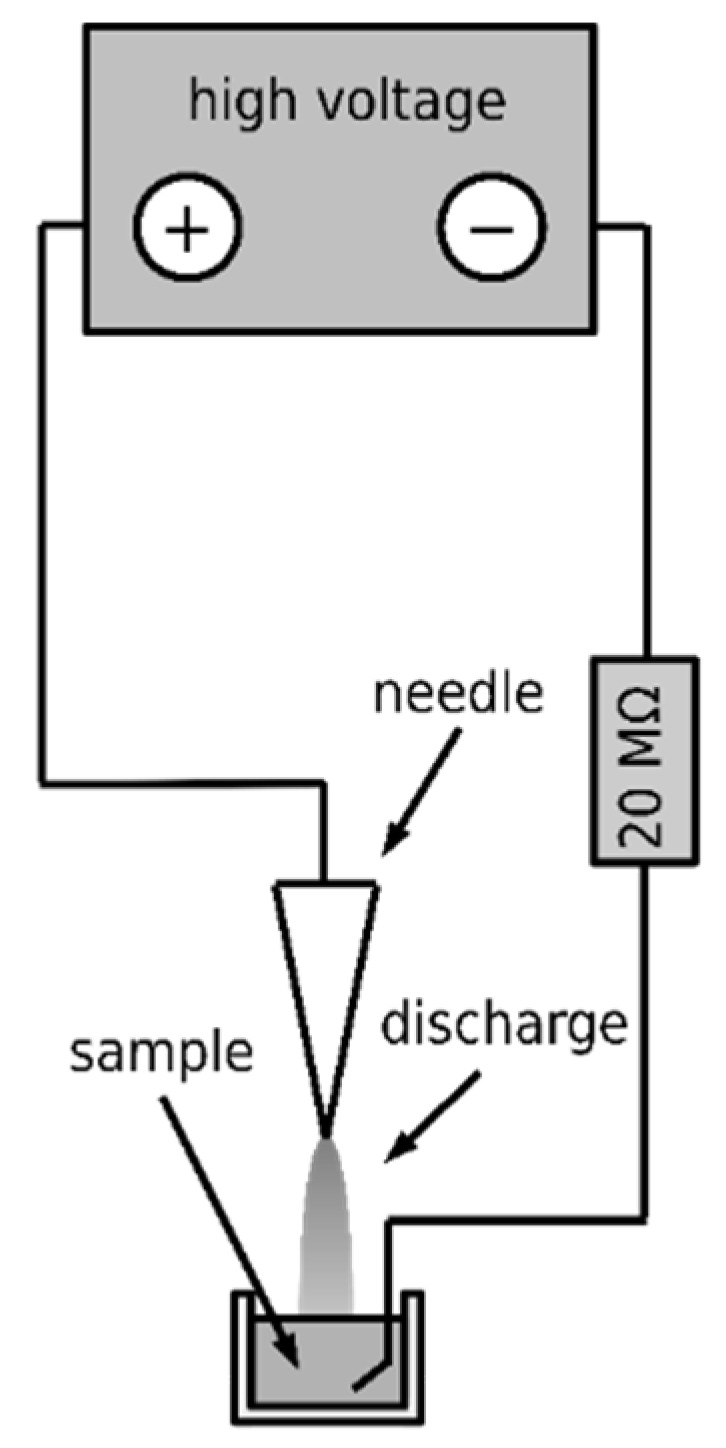
Scheme of the positive discharge.

**Figure 3:**
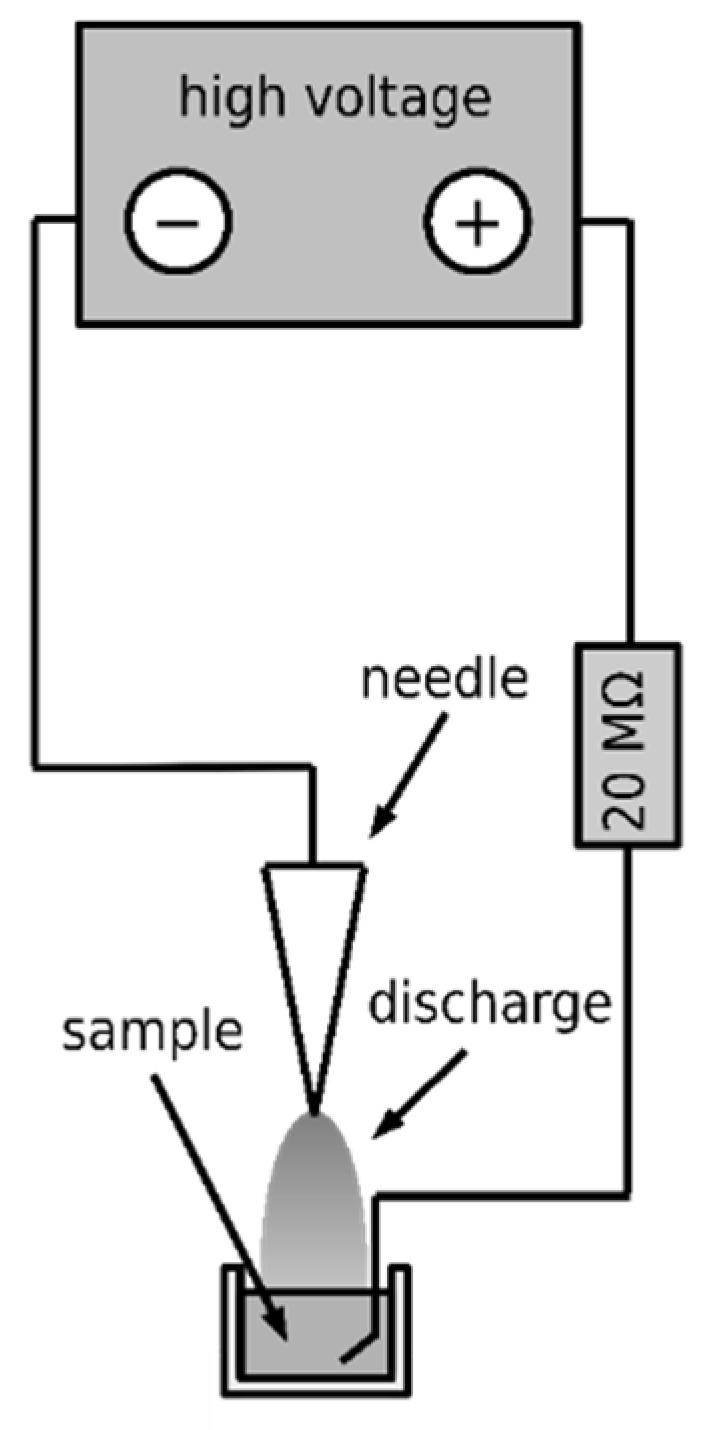
Scheme of the negative discharge.

**Figure 4:**
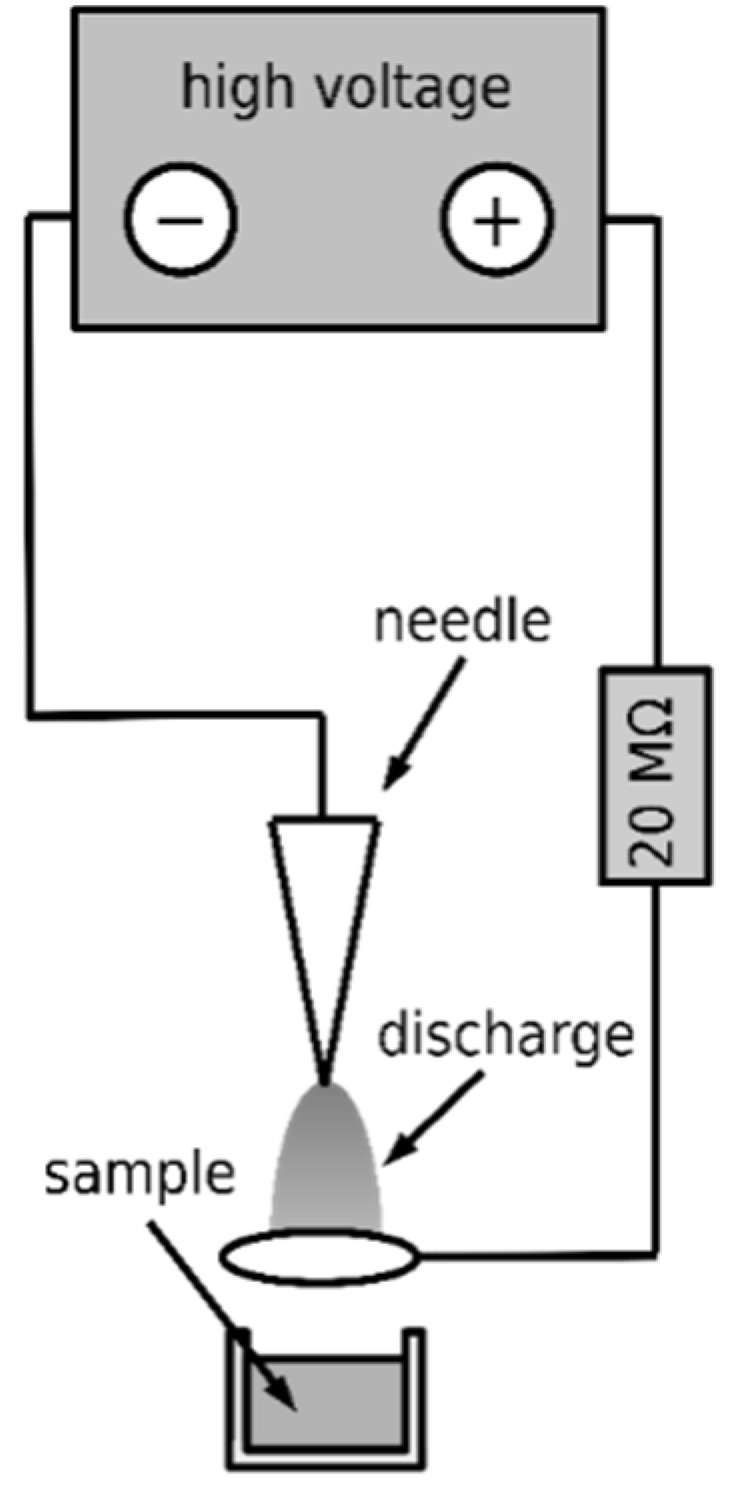
Scheme of the axial discharge.

Three types of DC corona discharges were employed to produce the non-thermal plasma, namely the positive discharge, negative discharge, and axial negative discharge. An intradermal medical needle was used as the working electrode in all cases, and a platinum wire was used as the ground electrode. An annular electrode was used as a ground in the case of axial discharge. Diagrams of their electrical connection are shown in Figure 2, Figure 3, and Figure 4, respectively. The voltage source HT 2103 (Utes Brno, Czech Republic) was used, and the discharges were stabilized by an inserted resistor *R* = 20 MΩ. The positive discharge was operated at voltage *U* = 9 kV and current *I* = 300 µA, the negative one at *U* = 9 kV and current *I* = 350 µA, and the axial one at *U* = 5 kV and *I* = 150 µA. In the case of the axial discharge, the current passed outside the exposed sample, on which only the generated active particles fell. The appearance and properties of individual discharges were described in [14].

### 2.2. Preparation of Plasma-Activated Water

Aliquots of 1.5 mL of bottled drinking water (Rosana, Czech Republic) were activated by exposure to positive, negative, and axial discharges, respectively. Different exposure times were used, respecting the different efficiencies of the individual discharges: 2 min for the positive, 8 min for the negative, and 75 min for the axial discharges. Details are given in the Results in Table 1. The achieved pH and concentrations of active components, namely nitrate, nitrite, and hydrogen peroxide, were estimated using Quantofix test strips (Macherey-Nagel, Düren, Germany).

### 2.3. Preparation of Infectious Larvae

*Schistosoma mansoni* cercariae were obtained from an infected intermediate host, the freshwater snail *Biomphalaria glabrata,* kept in permanent laboratory life-cycle. The required number of infected snails was transferred to a beaker with bottled drinking water (Rosana, Praha, Czech Republic), previously proven as optimal for the cultivation of the snails used, and placed under a light source, where cercariae were released within 30 min. Water with cercariae was sieved (1 mm mesh) to remove large organic parts (e.g., snail feces or plant food traces); cercariae were subsequently concentrated in the upper part of a darkened narrow neck beaker using positive phototaxy. The number of cercariae in 1 mL was assessed using binocular stereomicroscope, adjusted to approximately 50 cercariae per mL, and aliquots of 1 mL of this suspension were transferred to 5 mL bowls to be exposed to plasma.

*Schistosoma mansoni* miracidia were obtained from previously infected mice: immersing mice in water containing cercariae accomplished the infection; afterwards, the mice were kept under standard conditions. After 6 weeks, necessary to complete the life-cycle of *Schistosoma*, the mice were necropsied, and the livers were homogenized in drinking water. As in the previous case, the homogenate was placed under a light source, miracidia floated to the surface and were collected. Aliquots of the miracidia suspension were adjusted to samples with a volume of 1 mL containing approximately 20 miracidia and transferred to 5 mL bowls to be exposed to plasma.

The experiments including the use of the experimental animals for the present study were approved by the Ministry of Education, Youth and Sports of the Czech Republic (Approval Number MSMT-7063/2017-2). All the animals used in the study were maintained by certified persons (Certificate Number CZ 02627) in specifically accredited laboratories (Accreditation Number 70030/2013-MZE-17214), both issued by the Ministry of Agriculture of the Czech Republic.

### 2.4. Infectious Larvae Inactivation by Plasma

The above-described samples containing cercariae were exposed to the positive, negative, and axial discharges, respectively. Each suspension was exposed for one minute, observed and evaluated with a stereomicroscope (BEL Engineering, Monza, Italy), and re-exposed for another minute. This process was repeated until all cercariae were killed. The sample temperature reached maximal 35 °C as determined by an infrared thermometer, and its evaporation was negligible. The observed cercariae were counted and, according to the degree and nature of their damage, classified into eight categories (types), as summarized in Table 2. The whole procedure was repeated three times; the numbers of cercariae displaying particular types of damage were averaged, rounded to whole individuals, and are summarized in the Results. To rule out the possibility that the tail drop (Type VIII) was caused mechanically, the cercaria suspension was mixed for 10 min in a vortex mixer (Grant Instruments, Shepreth, UK) at ca. 1000 rpm, and evaluated as above.

In addition, the viability of cercariae was determined by staining them with methylene blue. This test is based on the fact that methylene blue penetrates only dead cells and stains them blue, while this stain does not penetrate living cells, so they remain unstained. Cercariae were obtained as described above under Preparation of Infectious Larvae. Aliquots containing approximately 20 cercariae were exposed to the positive or negative discharge for 1, 4, and 8 min. All samples were stained with methylene blue (0.15% in PBS, Sigma-Aldrich, St. Louis, MO, USA) for 15 min, then the non-absorbed methylene blue was washed with phosphate buffer, and the samples were evaluated using a stereomicroscope and photographed under the optical microscope Eclipse E200 (Nikon, Tokyo, Japan).

Miracidia suspensions were exposed to the positive, negative, and axial discharges, respectively. Each suspension was exposed for one minute, observed and evaluated with a stereomicroscope, and re-exposed for another minute. This process was repeated until all miracidia were evaluated as dead. The observed miracidia were counted and, according to the degree and nature of their damage, classified into 5 categories (types), as summarized in Table 3. The whole procedure was repeated three times, and the numbers of miracidia displaying particular types of damage were averaged, rounded to whole individuals, and are summarized in the Results.

### 2.5. Inactivation of Schistosoma Infectious Larvae in PAW

Aliquots of 10 μL suspensions containing approximately 50 cercariae or 20 miracidia were added to 1 mL of freshly activated water. Their damage was then evaluated by stereomicroscopic observation in the same manner as the samples directly exposed to plasma (see above).

## 3. Results

### 3.1. Direct Action of Discharges on Cercariae

**Figure 5:**
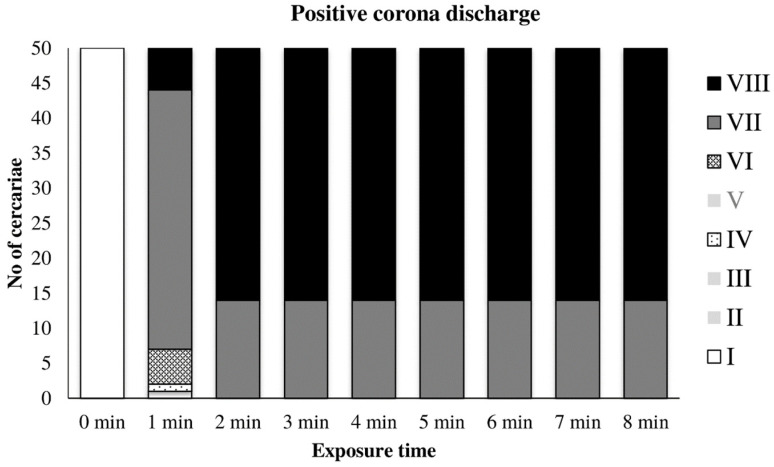
Inactivation of cercariae by positive discharge.

The graph in Figure 5 shows the action of the positive discharge on cercariae. The empty column corresponds to the unexposed control with intact cercariae. After 1 min of exposure, conspicuous damage of the cercariae was apparent, whereas only inactivated cercariae with no signs of life and without tails (Types VII and VIII) were present. No tail loss was observed after mechanical vortexing of the cercariae suspension.

**Figure 6:**
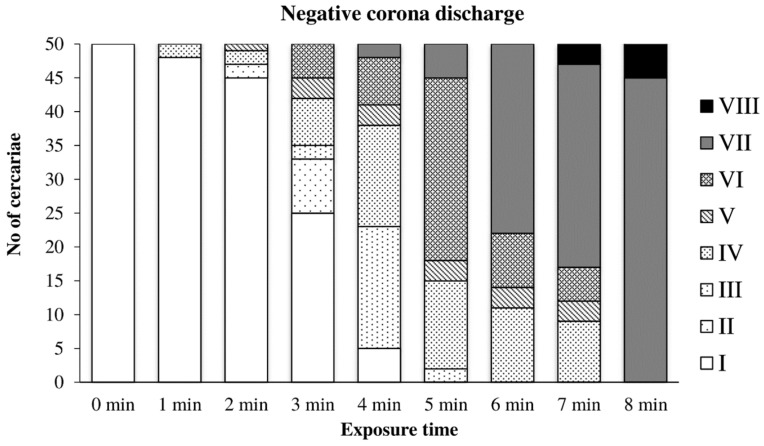
Inactivation of cercariae by negative discharge.

The graph in Figure 6 shows the gradual inactivation of cercariae by the negative discharge. A significant inactivation occurred later, after 3–4 min of exposure. Cercariae with no signs of life (Type VII) were observed after 4–5 min. Tail loss (Type VIII) occurred after 7 min, and complete inactivation occurred after 8 min of exposure.

The effect of axial discharge was considerably lower than that of other discharges. Only minor Types II and III damage was observed after exposure for 30 and 60 min. The inactivation of cercariae was observed after a long exposure of 75 min. Due to this negligible effect, the time dependence is not presented here in extenso.

### 3.2. Effect of PAW on Cercariae

Table 3 summarizes the estimated composition of pre-activated water (PAW) prepared by various discharges. Exposure times causing complete inactivation of cercariae were chosen for individual discharges, i.e., 2 min for the positive, 8 min for the negative, and 75 min for the axial discharges. In addition, PAW exposed for 30 min to the positive and negative discharges was prepared.

**Figure 7:**
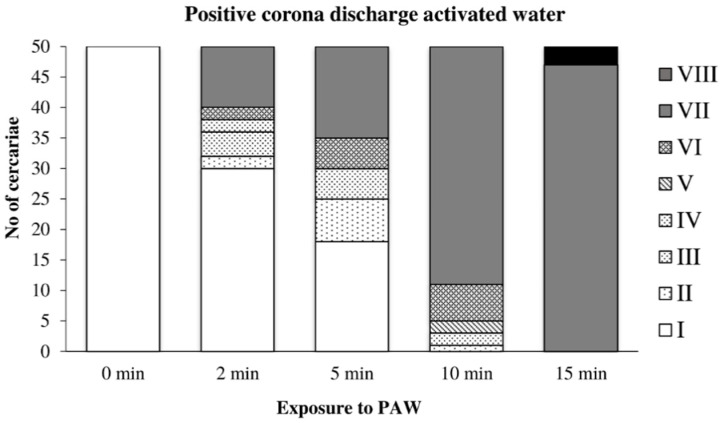
Inactivation of cercariae by the positive discharge-activated water.

The action of water activated by the positive discharge on cercariae is shown in Figure 7. The first signs of damage appeared after two minutes of exposure to this PAW; after 10 min, no cercaria showed any signs of active movement; after 15 min, all cercariae were inactivated. The action of this PAW caused a significantly lower incidence of tail dropping than the direct action of the positive discharge.

**Figure 8:**
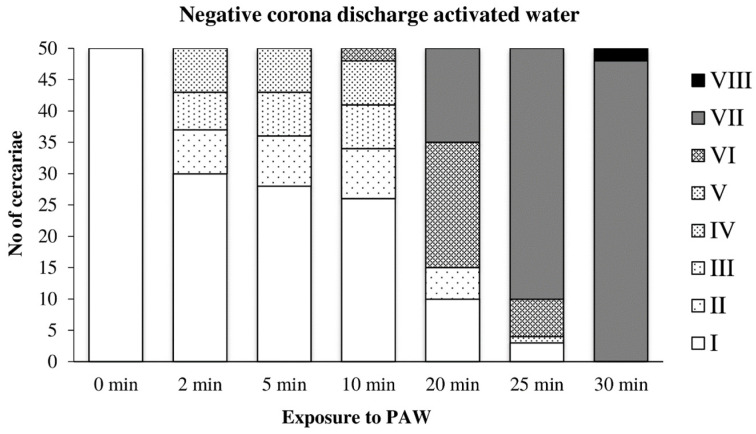
Inactivation of cercariae by the negative discharge-activated water.

Cercariae transferred to water activated by the negative discharge (Figure 8) were partially able to adapt to the adverse conditions and showed only slight damage after 10 min of exposure. After 25 min, very few cercariae with normal movement were observed, and the complete inactivation occurred after 30 min. Detached tails were found only rarely.

**Figure 9:**
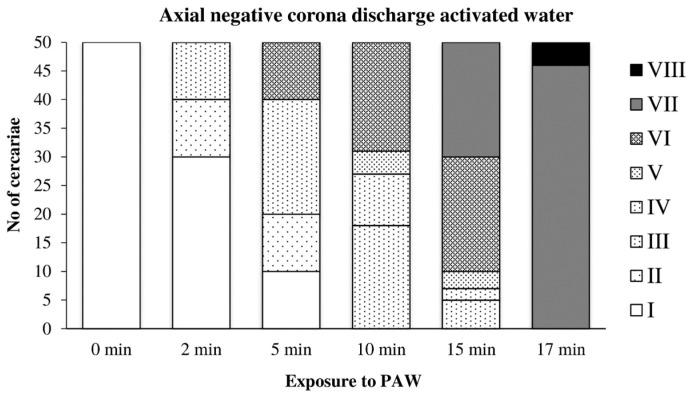
Inactivation of cercariae by the axial discharge-activated water.

In water activated by the axial discharge, the cercariae were significantly damaged after only five minutes of exposure; their movement was significantly limited after 10 min. After 17 min, they were completely inactivated (Figure 9).

### 3.3. Microscopic Observation of Exposed Cercariae

**Figure 10:**
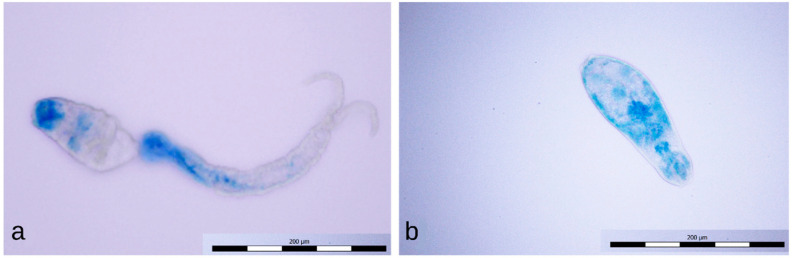
Partially stained cercaria (**a**) and dead cercaria without a tail stained with methylene blue (**b**).

**Figure 11:**
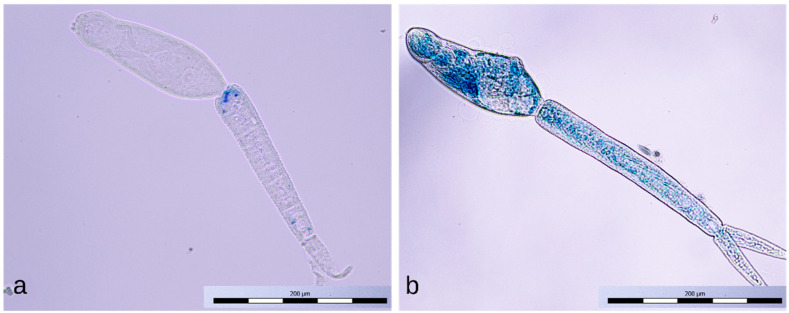
Intact cercaria (**a**) and dead cercaria stained with methylene blue (**b**).

Cercariae exposed for 1 min to the positive discharge were usually stained only partially with methylene blue (Figure 10a); sometimes, the tail detachment (Figure 10b), typical of longer exposures to this discharge, had already occurred. Cercariae exposed to the negative discharge for 1 and 4 min remained native (unstained, Figure 11a), while samples exposed for 8 min contained only inactivated stained cercariae (Figure 11b).

### 3.4. Direct Action of Discharges on Miracidia

**Figure 12:**
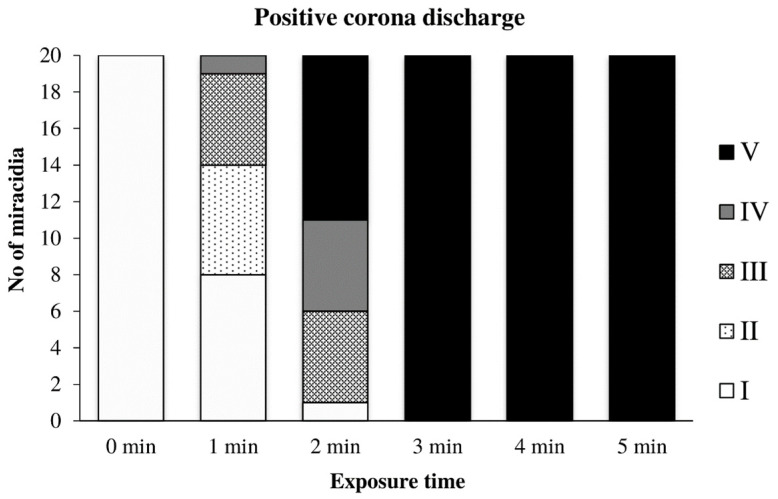
Inactivation of miracidia by the positive discharge.

The effect of the positive discharge on miracidia depending on the exposure time is summarized in Figure 12. It was apparent that the movement of miracidia was significantly affected already during the first minute of exposure; several moving miracidia were still visible at the second minute. Complete inactivation of the suspension with approximately 20 miracidia occurred after 3 min of exposure.

**Figure 13:**
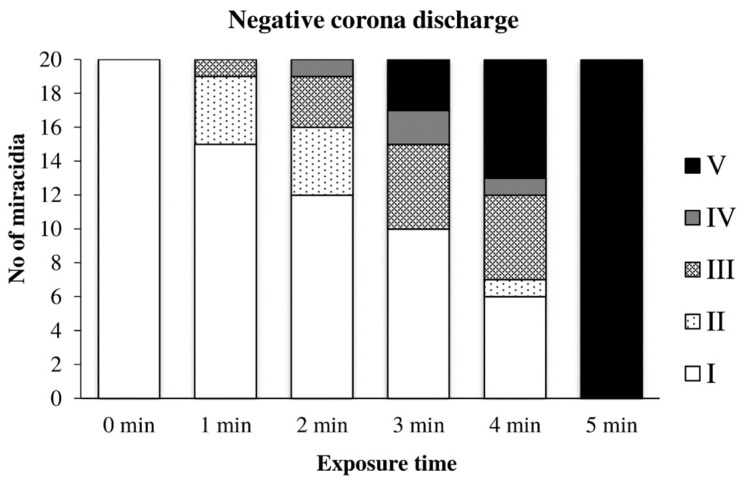
Inactivation of miracidia by the negative discharge.

Figure 13 shows the effect of the negative discharge on miracidia. Between the first and fourth minutes, a transitional area can be seen where miracidia tried to adapt and withstand the adverse conditions. However, after five minutes of exposure, they no longer showed any signs of life.

The axial discharge inactivated the miracidia suspension only after a very long exposure lasting 75 min, similar to that described under Section 3.1 for cercariae. Therefore, the observations of the inactivation process are not recorded in the graph. From this experiment, only a weak inactivating effect of this discharge on the miracidia of the genus Schistosoma was found.

### 3.5. Effect of PAW on Miracidia

**Figure 14:**
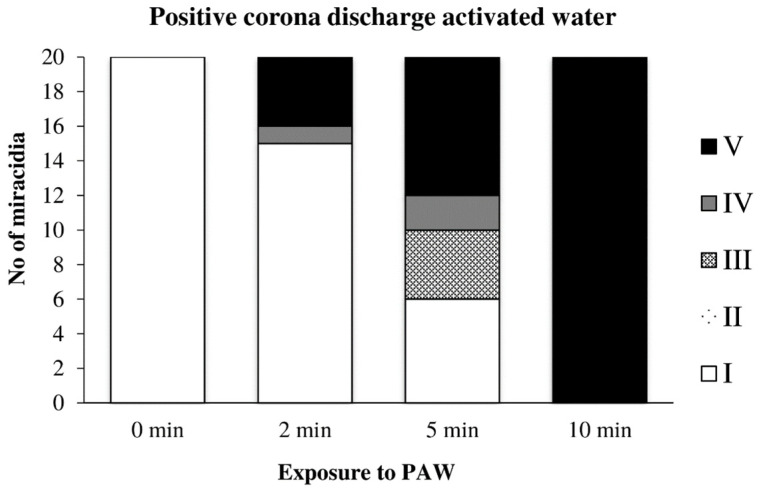
Inactivation of miracidia by water activated with the positive discharge.

Water previously activated by the positive discharge caused a noticeable inactivation of miracidia after only 2 min of action, and after 10 min, their inactivation was complete (Figure 14).

**Figure 15:**
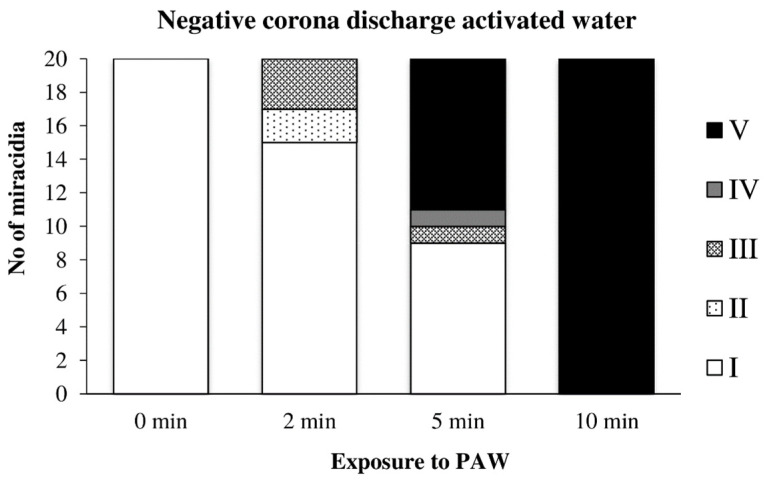
Inactivation of miracidia by water activated with the negative discharge.

Water previously activated by the negative discharge exhibited a similar, perhaps only slightly slower effect, as in the previous case (Figure 15).

**Figure 16:**
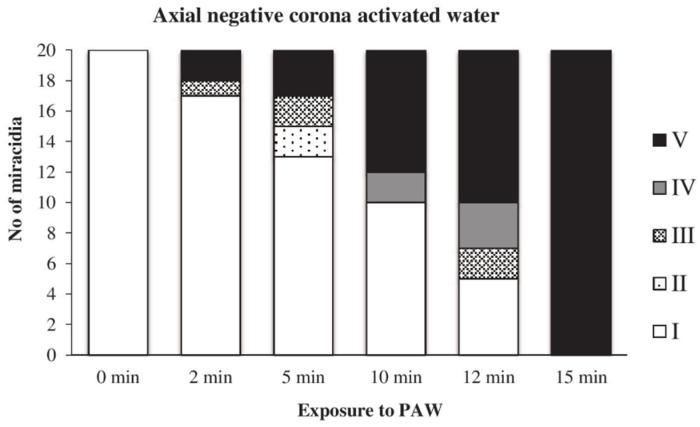
Inactivation of miracidia by water activated with the axial discharge.

The action of water activated by the axial discharge was even slower than in the previous two cases; this PAW caused the complete inactivation of miracidia after 15 min of exposure (Figure 16).

## 4. Discussion

In the previous study of [40], the complete inactivation of cercariae was not achieved even after ten minutes of exposure. However, the experimental conditions used were somewhat different from our report: *Schistosoma japonicum* and the dielectric barrier discharge were employed. We do not assume that our somewhat different results would be due to a different schistosome species, whose properties are unlikely to differ much. The different efficiency of inactivation is probably due to the different efficiency of the discharge used, caused by different mechanisms of its action. This is apparent from a comparison of the efficiencies of different types of corona discharge, summarized in the Results. The positive discharge appeared as the most effective, causing the complete inactivation within 2 min of exposure. The negative discharge needed 8 min for the same effect, but the efficiency of axial discharge was negligible. In addition, only after exposure to the positive discharge the tail detachment was observed. Inactivation of cercariae by plasma-activated water gave similar results, with PAW activated by a positive discharge appearing to be the most effective. PAWs activated by negative and axial discharges showed somewhat weaker, but remarkable and comparable efficiencies.

For the first time, the results of the inactivation of miracidia by low-temperature plasma are presented here. The direct action of discharges displayed similar trends as for cercariae: the positive discharge inactivated miracidia within 2–3 min, and the negative one after five minutes of exposure. The axial discharge appeared as almost ineffective. Concerning PAW, it inactivated miracidia after 10 min of action, both after activation with positive and negative discharge. The effect of water activated by axial discharge was comparable, although somewhat slower.

If completely inactivated, schistosomes were indeed dead, as demonstrated by determining their viability after methylene blue staining. This finding is significant as it denies the possibility of recovery and subsequent infection by partially inactivated schistosomes.

The observation of exposed cercariae by electron microscopy provided preliminary results indicating different mechanisms of action for different discharges. These findings are not reported here, but we are working on a more detailed study confirming this assumption, which will be published separately.

This holds also for the explanation of the mechanisms involved in the schistosome inactivation. The chemical processes taking place in exposed water are relatively complex, including various active components’ production, which have not yet been fully explained. They have been discussed and summarized in many contributions, such as the reviews of [29,31], and we contributed to this discussion in [28,41]. The discharges used differ by the rate of reactive particles generated. For the most effective positive discharge, atomic oxygen, nitrogen, and OH^˙^ radicals were generated at higher amounts than in the less effective negative one. The axial discharge was similar to the negative one, but it burned over the sample only and not the sample directly. We believe that there is no place in this work for a more detailed analysis of this voluminous issue.

The results presented in this paper are only pilot studies and apply only to exposures of small volumes. For their practical use, further studies would be needed, using, for example, the multi-electrode discharge arrangement or dielectric barrier type discharges capable of hitting a larger volume of exposed liquid.

## 5. Conclusions

The low temperature plasma produced by corona discharges, as well as water activated by these discharges (PAW) inactivate the cercariae and miracidia of *Schistosoma mansoni*. The efficiency of this inactivation depends strongly on the nature of the discharge: the positive discharge works within 2–3 min of exposure and the negative one within 5–8 min. The direct action of the axial discharge is almost ineffective. Similar effects were observed for the PAW, except for the PAW activated with the axial discharge, which inactivated cercariae and miracidia after 17 and 15 min, respectively. Preliminary microscopic investigation implies different mechanisms of inactivation for particular discharge types.

## Figures and Tables

**Table 1:** Properties of pre-activated water (PAW).

	Exposure Time (min)	pH	NO_3_^−^ (mg·L^−1^)	NO_2_^−^ (mg·L^−1^)	H_2_O_2_ (mg·L^−1^)
unexposed	0	7	<15	0	0
positive discharge	2	6	250	0	25
	30	4	>500	40	>100
negative discharge	8	6	250	0	10
	30	4	>500	40	10
axial discharge	75	6	500	40	25

**Table 2:** Types of cercariae damage.

Damage Type	Description
Type I normal movement	Fast swimming employing tail movement
Type II resting position	Floating in a vertical position without movement
Type III sinking to the bottom	No movement; sinking to the bottom
Type IV crawling at the bottom	Spasmodic motion at the bottom
Type V normal movement with tail detachment	Active crawling of the cercarial body at the bottom
Type VI agony	Occasional twitches at the bottom
Type VII death	No activity
Type VIII death with tail detachment	No activity; the tails detached

**Table 3:** Types of miracidia damage.

Damage Type	Description
Type I normal movement	Swimming in the water
Type II slow motion	Floating with active movement rarely seen
Type III crawling at the bottom	Active movement at the bottom
Type IV agony	Occasional twitches at the bottom
Type V death	No activity

## Data Availability

No additional data were created or analyzed in this study. Data sharing is not applicable to this article.
